# Effects of Inner-Row Ground Management on the Volatomics of ‘Cabernet Sauvignon’ Grapes and Wines in the Region of the Eastern Foothills of the Ningxia Helan Mountains in Northwest China

**DOI:** 10.3390/foods12132472

**Published:** 2023-06-23

**Authors:** Fei He, Meng-Bo Tian, Wei-Peng Duan, Wei-Ming Yang, Xue Mao, Jun Wang, Chang-Qing Duan

**Affiliations:** 1 Center for Viticulture and Enology, College of Food Science and Nutritional Engineering, China Agricultural University, Beijing 100083, China; 2 Key Laboratory of Viticulture and Enology, Ministry of Agriculture and Rural Affairs, China Agricultural University, Beijing 100083, China; 3 Department of Grape and Wine Engineering, College of Food Science and Engineering, Gansu Agricultural University, Lanzhou 730070, China; 4 Gansu Key Laboratory of Viticulture and Enology, Lanzhou 730070, China; 5 E&D Center of Wine Industry in Gansu Province, Lanzhou 730070, China; 6 Chateau Zhihui Yuanshi, Yinchuan 750026, China

**Keywords:** crop covering, mulching, grape, wine, aroma compounds

## Abstract

This two-consecutive-year study aimed to evaluate the effects of ground management methods on the volatomics of ‘Cabernet Sauvignon’ grapes and wines in Northwest China, in which inner-row crop covering with purslane (GRASS) and mulching with black plastic film (FILM) treatments were carried out, respectively. Compared with clean tillage (CK), the GRASS and FILM treatments changed the microclimates of grapevine fruit zones and rhizospheres, which delayed the ripening of grape berries and affected the accumulation of aroma substances in the mature grapes effectively. GRASS increased the concentration of terpenes and C_13_-norisoprenoids in berries and gave more floral, fruity, and caramel fragrances to wines, while FILM had the opposite effect of significantly increasing the synthesis of C_6_/C_9_ compounds and brought more green leaf flavors, showing that inner-row purslane covering is a potential and stable viticultural practice to improve the wine quality in this booming wine region.

## 1. Introduction

As a group of important flavor substances, aroma compounds directly affect the sensory quality of grape berries and wines. Until now, hundreds of grape and wine volatile compounds have been identified, mainly including alcohols, aldehydes, acids, esters, terpenes, C_13_-norisoprenoids, pyrazines, and sulfur compounds [1]. Among them, the volatiles in wines originating from grape berries were classified as varietal aromas, the composition and concentration of which were affected by many factors such as grape variety, maturity, climate, soil, and viticultural management [2,3,4].

Generally, the French word “terroir” is a technical term in wine science, which refers to the combination of natural environmental conditions (including the climate, terrain, soil, etc.) and anthropic factors (including viticulture, vinification, aging, etc.) that shape the styles and characteristics of wines in a particular region [5,6]. Viticultural treatment is one part of the generalized “terroir”, but sometimes it is not strong enough to shake the decisive influence of the geographical, geological, climatic, and meteorological conditions of one region; however, it is beneficial for wine quality improvement. Choosing an appropriate viticultural method in a certain geographical and climatic environment is especially important to produce wines with good quality [7].

Ground management is an important part of viticultural treatments, mainly including clean tillage, mulching, crop covering, and no tillage [8]. Crop covering refers to the artificial cultivation of annual or perennial weeds within and between rows of grapevines, or in the whole garden [9]. Moreover, grape growers mainly cultivate natural grass and remove malignant weeds, which is of great significance to the environmental protection and sustainable development of vineyards [10]. Some previous studies showed that crop covering improved the micro-environment of the fruit zone and root zone of grapevines, thereby contributing to the growth and development of vines [11]. For example, in some regions with a temperate marine climate or Mediterranean climate, the soils are fertile and the grapevines cultivated there usually have strong vigor and high yield. Thus, crop covering limits the excessive vegetative growth of grapevines and improves the quality of grape berries and resultant wines [12]. However, other studies showed that crop covering in some semi-arid environments affects the soil moisture, root penetration, and grape yield, competes with grapevines for water and nutrition, and increases the rodent population and the cost of vineyard management, thus causing some adverse effects [13].

Mulching is another common method of vineyard ground management. The microclimate and soil conditions of grapevines can be modified by properly laying organic or inorganic mulch on the surface of the ground within or between rows of grapevines. The mulching method increases soil temperature, retains water and fertilizer, reduces weeds, reduces the occurrence of diseases and pests, etc., which promotes the growth and development of grapevines to a certain degree and improves the quality of grape berries and resultant wines [14]. By selecting different mulch materials, covering areas, times, and duration, the growth of grapevines and fruit quality are affected differently [15]. Until now, the research on crop covering and mulching in vineyards mostly focused on their effects on the soil structure, water utilization, and vine growth [16,17], while the investigations into the effects on the flavors of grape berries and wines were still very limited.

The region of the Eastern Foothills of the Ningxia Helan Mountains is located in northwest China, which has a temperate continental monsoon climate. In summer when grape berries develop rapidly, this region often experiences problems of too strong light, too high temperature, and too much heat accumulation, which results in too fast fruit development and insufficient accumulation of flavor substances; this affects the further improvement in the wine quality there [18]. As this region is located between the Yellow River alluvial plain and the Helan Mountain alluvial fan, the soil texture is mainly sandy soil and gravel, and the strong ground reflection created by this soil type further aggravates these problems [19]. In the present study, the major wine grape variety ‘Cabernet Sauvignon’ (*Vitis vinifera* L. cv.) in this region was selected, and clean tillage (CK) was taken as the control. The ground management methods of inner-row crop covering with purslane (GRASS) and inner-row mulching with black plastic film (FILM) were carried out, and their effects on microclimate, fruit development, aroma compound accumulation in grape berries, and resultant wines were studied for the two consecutive years of 2016 and 2017. This study is expected to provide some support for the development of ground management methods suitable for the local conditions of this new, important, and fast-growing wine region in China.

## 2. Materials and Methods

### 2.1. Chemicals

Acetonitrile, formic acid, and methanol were purchased from Fisher Company (Fairlawn, NJ, USA). *D*-(+)-dextronic acid δ-lactone was purchased from Sigma-Aldrich (St. Louis, MO, USA). The Cleanert^®^ PEP-SPE resin (150 mg/6 mL) was obtained from Bonna-Agela (Wilmington, DE, USA).

The standards (purities > 95%) used for identification and quantification of the volatile compounds 1-hexanol, (*E*)-2-hexen-1-ol, (*Z*)-2-hexen-1-ol, (*E*)-3-hexen-1-ol, (*Z*)-3-hexen-1-ol, hexanal, (*E*)-2-hexenal, 1-nonanol, furfural, hexanoic acid, limonene, *D*-limonene, myrcene, linalol, *α*-terpineol, 4-terpineol, *β*-citronellol, citral, geranylacetone, geraniol, nerol, *cis*-furan oxidin, spirulina, (*Z*)-orange sterol, (*E*)-*β*-damascenone, (*E*)-*β*-ionone, and 4-methyl-2-pentanol were all supplied by Sigma-Aldrich (St. Louis, MO, USA). The deionized water (<18 WΩ resistance) was obtained through a Milli-Q Element water purification system (Millipore, Bedford, MA, USA). All the other chemicals or reactives without especial mention were purchased from Beijing Chemical Works (Beijing, China).

### 2.2. Plant Material

The two-successive-year (2016 and 2017) study was performed in a commercial vineyard (38°35′37″ N–106°1′30″ E, elevation 1140 m) at Chateau Zhihui Yuanshi in Yinchuan City of Ningxia Hui Autonomous Region, which belongs to the region of the Eastern Foothills of the Ningxia Helan Mountains in Northwest China. The vineyard is about 6.35 hectares, with a semi-arid continental climate. The soil of the vineyard is the typical sandy gravel structure soil of this region. The own-rooted ‘Cabernet Sauvignon’ vines were first planted in the year 2013 and were trained to the modified vertical shoot-positioned spur-pruned cordon system (M-VSP). The row and vine spacing were 4.0 × 1.2 m, respectively, with the orientation of north–south. The fruit zone was about 80 cm above the ground.

### 2.3. Experimental Design and Sampling

In the vineyard, 30 rows of grapevines with similar growth vigor were selected. Three ground management methods were chosen in this study, with 10 rows for each treatment and about 180 vines for each row: clean tillage (CK) was used as the control; inner-row purslane (*Portulaca oleracea*) covering (GRASS) and black plastic film mulching (FILM) were selected as treatments in the two consecutive years of 2016 and 2017, as shown in the Supplementary Materials, Figure S1.

Purslane is a dicotyledonous plant and is one of the local wild weeds with relatively strong vigor. Moreover, this weed usually grows low to the ground, and its height is rarely higher than 15 cm in the studied region. In the GRASS treatment, the seeds of purslane were sown manually in the 1.2 m wide strip directly under the grapevines in the spring (in April, after the grapevines were unearthed) of the year 2016. Since purslane grows vigorously in the local area and has strong seed-bearing and reproduction abilities, it can become the dominant weed without sowing in the following years. In the autumn (November), the purslane plants gradually dried and died and were removed later when the grapevines were buried.

For the FILM treatment, black plastic mulch with a thickness of 0.10–0.12 mm and a width of 2.4 m was used. After the grapevines were unearthed, the mulch was laid in the rows to cover the ground. Both sides of the mulch were buried and fixed with soil, which can be recycled before the grapes were pruned and buried in the autumn. In our experience, such mulch can be used for three years at least.

The samples were collected at the commercial harvest stage (E-L 38), when the total soluble solids (TSSs) concentration of grape berries reached about 24–25° Brix and the titratable acid (TA) concentration reached 5–6 g/L (as tartaric acid equivalents). The sampling was carried out randomly with an S-shaped route. For each treatment, a total of 600 berries from more than 100 clusters from more than 20 vines were sampled as 1 replicate and 3 replicates were collected. After sampling, for each replication, 300 berries were randomly selected to measure the physicochemical parameters of the grape berries, and the remaining berries were frozen immediately with liquid nitrogen and stored at −80 °C for the subsequent aroma analysis.

### 2.4. Mesoclimate and Microclimate Data

The mesoclimate data, including the sunshine duration, average daily temperature, and rainfalls in the whole growing season of grapevines (from 1 April to 30 November) in 2016 and 2017 were obtained from the China Meteorological Data Service Centre (http://cdc.cma.gov.cn/) (accessed on 25 May 2022). The weather station is about 20 km away from the vineyard.

The microclimate was monitored in the central row of each treatment. The temperature and humidity sensor (S-THB-MOO2, Onset, Bourne, MA, USA) and the PAR sensor (S-LIA-M003, Onset, Bourne, MA, USA) were installed parallel to the cordon at the fruit zone. In addition, the soil moisture was monitored via a soil humidity sensor (S-TMB-M002, Onset, Bourne, MA, USA), which was buried 40 cm under the ground surface. The meteorological data were recorded at five-minute intervals via a HOBO micro station logger (H21-002, Onset, Bourne, MA, USA).

### 2.5. Phenological Observation and Yield Measurement

The growing seasons and phenological phases of grapevines from the three treatments were monitored and recorded. The photos of grape clusters at five key developing stages, namely, pea size (E-L 31), veraison 5% (E-L 35), veraison 100% (E-L 36), 20 days after veraison 100% (E-L 37), and the commercial harvest stage (E-L 38), were collected. At harvest, ten randomly sampled clusters from each treatment were weighed and the average cluster weight were calculated, and the number of clusters from ten vines was counted for each treatment to calculate the productivity and yield.

### 2.6. Industrial Vinification

After sampling, the rest of the grapes in each treatment area and in good sanitary condition were manually harvested. Then, the harvested grapes were transported to the winery with clean plastic baskets immediately. Before destemming, the grapes were cluster selected by hand to eliminate the poor matured clusters and impurities. Finally, after destemming, the grapes were further berry selected to ensure quality and homogeneity of the grape berries.

After crushing, the must was transferred into a 100 hL stainless steel fermenter equipped with a cold jacket temperature control system, and the loading volume was about 75–80% of the capacity. For each treatment, two fermenters were used as replicates. At the same time of must pumping, 40 mg/L of sulfur dioxide, 20 mg/L of pectinases (Optivin^®^, Sydney, Australia), 2.5 g/L of tartaric acid, and 100 mg/L of tannin (Protanin R, Lamothe-Abiet, France) were added into the must on line. After cold maceration at 6–8 °C for 5 days, 200 mg/L of Lalvin strain D254 yeast (Lallemand Inc., Blagnac, France) was added to the must to start the alcoholic fermentation (AF) process, which was conducted at a relatively constant temperature of 22–25 °C. During this process, skin caps were punched down three times a day and the pumping over technique was adopted at the same interval for 20 min each time. When the reduced sugar of the wines reached about 4 g/L and never changed, AF was supposed to be finished, and 10 mg/L of commercial malolactic bacteria (Lalvin 31, Lallemand Inc., France) was added to start the malolactic fermentation (MF) process. Four days after inoculation, wines were separated from pomaces, and MF was conducted at a relatively constant temperature of 24–26 °C. When MF ended, 60 mg/L of sulfur dioxide was added to the wines. Wine samples were collected at the end of AF and MF, respectively, and were then filtered, bottled and stored at −40 °C for further analysis.

### 2.7. Analysis of Berry and Wine Physiochemical Parameters

For each replicate of grapes, 300 berries were weighed, crushed, and pressed uniformly, and the obtained must was analyzed to determine the physiochemical parameters. TSSs (total soluble solids) was measured via a PAL-1 digital hand-held refractometer (Atago, Tokyo, Japan). TA (titratable acidity) was detected via titration with NaOH (0.05 M) to the endpoint of pH 8.2 and expressed as the tartaric acid equivalents. The pH values were measured via a Mettler LE438 pH meter (Mettler, Toledo, Switzerland).

The wine residual sugar, volatile acidity, and ethanol content were determined according to the methods of OIV. Wine total acidity (TA) was analyzed in the same way as TA in must. Before analysis, the carbon dioxide was extracted by using a degasser. Wine pH was determined by using a pH meter (Sartorius PB-10, Gottingen, Germany).

### 2.8. Extraction of Berry Aroma Compounds

Free and bound aroma compounds were extracted according to the method of Lan et al. [20] with slight changes. For each replicate, about 80 g of frozen berries were de-seeded under liquid nitrogen. Then, the de-seeded samples were grounded into powder with an addition of polyvinylpolypyrrolidone (1 g) and *D*-gluconic acid lactone (0.5 g).

The frozen powder was transferred into a 50 mL centrifuge tube, then melted and macerated at 4 °C for 2 h. The clean juice was obtained through centrifuging at 8000× *g* for 15 min. For free-form volatile compounds, 5 mL of clean juice was added to a 20 mL vial containing a magnetic stirrer with 10 μL of 4-methyl-2-pentanol (which was employed as the internal standard) and 1 g of NaCl. For bound-form volatile compounds, 2 mL of clean juice was added to Cleanert^®^ PEP-SPE resins, which had been activated with 10 mL of methanol and 10 mL of water. Then, the resins were washed with 2 mL of water and 5 mL of dichloromethane to remove the water-soluble compounds and free-form volatiles, respectively. The resins were eluted via methanol afterward and the methanolic substance was collected. The collected substance was concentrated to dryness via a rotary evaporator under a vacuum at 30 °C and was redissolved in 10 mL of citrate/phosphate buffer solution (0.2 M, pH = 2.5). After hydrolyzing under 100 °C for 1 h in citric acid in an encapsulated vial, 5 mL of the solution was added to a 20 mL vial containing a magnetic stirrer, 10 μL of internal standard (4-methyl-2-pentanol), and 1 g NaCl.

The volatile compounds from the grape and wine samples were extracted and concentrated via headspace solid-phase microextraction (HS-SPME), and all the samples were determined for triplicates, as described by Wang et al. [21].

### 2.9. GC-MS Analysis of Aroma Compounds in Grapes and Wines

The analysis of volatile compounds was performed on an Agilent 7890 gas chromatograph (GC) coupled to an Agilent 5975B mass spectrometer (MS), fitted with an HP-INNOWAX capillary column (60 m × 0.25 mm, 0.25 μm, J and W Scientific, Folsom, CA, USA). The GC-MS conditions were settled according to Wang et al. [21] with slight changes. Oven temperature began at 50 °C for 1 min, then increased to 220 °C at a rate of 3 °C/min and held at 220 °C for 5 min. The temperature of the ion source and quadrupole was set at 230 °C and 280 °C, respectively. High-purity helium was the carrier gas at 1 mL/min and the GC inlet was set in the splitless mode. The full scan mode was employed to collect electron ionization mass data from 30–350 u. The ionization voltage was set at 70 eV.

The identification of volatile compounds was conducted by using the automated mass spectral deconvolution and identification system (AMDIS), which matched the mass spectrum and RI with the reference standards in the NIST 11 MS database. The quantification of volatile compounds was conducted according to the corresponding external standards, as described by Wang et al. [21]. The concentrations of those volatile compounds without corresponding standards were estimated with the equations of standards having the same functional group and/or similar numbers of carbon atoms. The concentrations of volatile compounds were expressed as μg/L in wines and μg/kg in fresh berry weight.

### 2.10. Statistical Analysis

Statistical analysis of data was performed via SPSS 23.0 with three replications of the same sample, and analysis of variance (ANOVA) was used to determine the significance of the difference among samples at a significance level of *p* < 0.05. Figures were created using GraphPad Prism 8.0.2.

## 3. Results and Discussion

### 3.1. Vintage Meteorological Difference

As shown in the Supplementary Materials, Table S1, the 2016 and 2017 vintages experienced high temperatures and little rainfall in summer, which was typical of the semi-arid continental climate in the region of the Eastern Foothills of the Ningxia Helan Mountains. The air temperatures, sunshine duration, and amount of rainfall were almost within the range of those between 2011 and 2020, as shown in the Supplementary Materials, Figure S2.

### 3.2. Microclimate Difference among Treatments

In 2016, compared with CK, the daily average temperature at the fruit zone of the GRASS treatment area at the pea size stage was decreased by 0.5–2.0 °C, while that of FILM treatment area was decreased by 0.3–1.0 °C, as shown in the Supplementary Materials, Figure S3. This phenomenon lasted until the end of veraison, but this cooling effect was poor in the fruit mature period. In 2017, such large temperature differences only occurred during veraison, and the differences were not obvious in other periods. The survey found that at about 2:00 pm in July and August when the temperature was relatively high, compared with CK, the temperature could be reduced by 3–5 °C for the GRASS treatment area and 2–3 °C for the FILM treatment area. For grapes, the optimum temperature for photosynthesis was 25–30 °C. When the temperature was higher than 35 °C, the stomata of leaves closed, and the photosynthesis and respiration dropped sharply. Moreover, when the temperature exceeded 45 °C for a while, leaves died, which resulted in a decline in grape maturity and quality [22]. Therefore, the fruit zone temperature could be appropriately reduced by the GRASS and FILM treatments, which could effectively solve the problem of extremely high temperatures. In one of our previous studies in the region of North Foothill of Tianshan Mountain, the inter-row black plastic mulching method decreased the low temperature (10–20 °C) duration, but it increased the high temperature (>30 °C) duration, which meant its effect might not be as good as the present one [15].

Different ground treatments also had certain effects on the air relative humidity at the fruit zone, as shown in the Supplementary Materials, Figure S3. In 2016, there were significant differences between the treatments at the pea size stage and veraison. Compared with CK, the air relative humidity of the GRASS treatment area was increased by 5–7%, and that of the FILM treatment area was increased by 2–5%. However, this effect was not obvious in 2017, which might be due to the reduced and concentrated rainfall during veraison. This result was also similar with one of our previous studies with inter-row peanut growing in Northwest China, which was even drier [9].

Compared with the pale-yellow ground surface after clean tillage, green grass or black mulch in the rows had selective absorption of light, causing a certain change in the light intensity reflected from the ground to the fruit zone, which was also the fundamental reason for the change in the air temperature and humidity in the treatment areas. The data from both of the two years showed that, compared with CK, the FILM treatment effectively reduced the light intensity of the fruit zone by 45–60%, and the GRASS treatment also reduced it by 10–20%, as shown in the Supplementary Materials, Figure S3. Sandy loam and light calcareous soil with gravel were the main soil types in this region, which had limited ability to absorb light and a strong ability to reflect light [23]. Therefore, in addition to raising the height of the fruit zone, the GRASS and FILM treatments provided alternative and effective ways to change the total radiant light intensity at the fruit zone.

These ground treatments also had diverse effects on soil water content of the grapevine rhizosphere (40 cm below the ground surface). Compared with CK, the soil water content of FILM was increased by 20–50%, while that of GRASS was decreased by 50–80%. This was because the plastic film used in mulching covered the ground surface, significantly reducing the soil evaporation, and thus played an important role in water conservation. Nevertheless, GRASS created water competition between weeds and grapevines, and the transpiration of weeds also reduced the water content in soils. In other warm and dry regions, such as Southern Australia, covering crops resulted in lower soil moisture, since the water supply was limited during the growing season and, therefore, the crops significantly competed with the grapevines for water [24]. Similar results were also obtained from reports of inter-row covering of crops in a sloping vineyard, in which it significantly reduced the average soil water content [25]. However, the soil type and regional climatic conditions greatly influenced the effect of covering crops on soil water content of the grapevine rhizosphere. Therefore, in a humid region with sufficient water supply for the vines and crops, the results might be minimal, or even the opposite [26,27].

Some previous studies showed that crop covering and mulching in vineyards not only changed the microclimate of the fruit zone, such as the light intensity, light quality, temperature, and humidity during berry development, but also modified the microenvironment conditions of the root zone, such as soil temperature and moisture, thus affecting the growth and development of the vines [28]. Other studies also showed that crop covering in orchards changed the light intensity and quality, as well as the temperature and humidity of the surrounding environment, thus affecting the physiology and morphology of plants [15]. Generally, our results were consistent with these reports.

### 3.3. Effects on Phenological Phases

**Figure 1:**
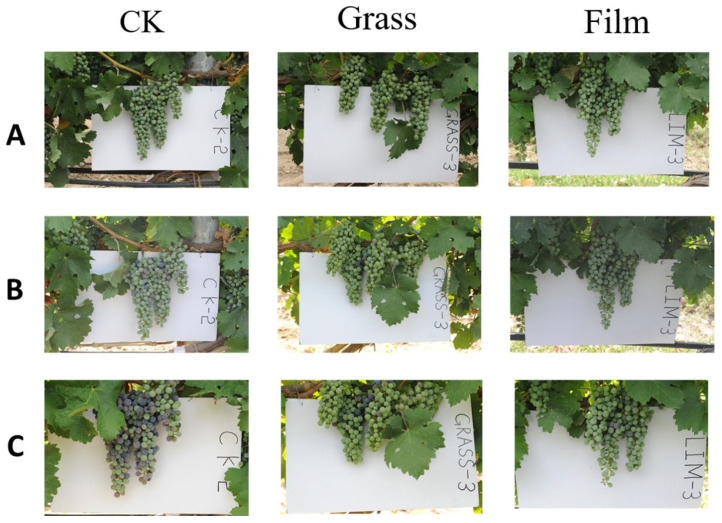
The influence of inner-row ground management methods on the veraison of ‘Cabernet Sauvignon’ grapes in the vintage of 2017. CK: clean tillage; GRASS: inner-row purslane covering; FILM: inner-row black plastic film mulching. (**A**) Photos of grape clusters with different treatments when the grapes of CK were at the developing stage of veraison 5%; (**B**) photos of the grape clusters with different treatments when the grapes of the GRASS treatment area were at the developing stage of veraison 5%; and (**C**) photos of grape clusters with different treatments when the grapes of the FILM treatment were at the developing stage of veraison 5%.

In both of the two vintages, the GRASS and FILM treatments regulated the phenological periods of grapevines, especially the veraison. The veraison of the CK, GRASS, and FILM methods lasted 14, 15, and 15 days in 2016, and 19, 16, and 11 days in 2017, respectively. Compared with CK, GRASS and FILM treatments delayed the beginning of veraison by 3 and 4 days in 2016, and 5 and 10 days in 2017, respectively, as shown in Figure 1. This might be due to the effects of GRASS and FILM treatments on grapevine microclimates, which affected the grapevines’ water and nutrition availability, as well as their metabolisms. In 2017, the annual rainfall was lower, but there were more concentrated rains during veraison, which accelerated the fruit development. However, in reports from other regions with quite different climatic conditions, especially where the whole growing season was longer, the development of the grape berries was not impacted significantly [9].

### 3.4. Effects on Grape Maturation and Yield

Compared with CK, the GRASS and FILM treatments increased the concentration of TSSs and decreased that of TA in mature grape berries in both of the two vintages, which was more obvious in 2016. Since GRASS and FILM modified the fruit-zone microclimate and the root-zone microenvironment, which improved the efficiency of leaf photosynthesis, the grape berries accumulated more sugar. As the purslane used in crop covering did not have nitrogen fixation capacity, the GRASS treatment led to competition between the purslane plants and the grapevines for water and nitrogen, which accelerated the decomposition of organic acids in grape berries, especially the malic acid, leading to a reduction in TA [29]. While the FILM treatment not only increased the soil water content but also changed the soil microbial colonies and nitrogen availability, it also caused a decline in TA [30]. Furthermore, in the two consecutive years, the 100-berry weight of the FILM treatment area was not significantly affected, while the GRASS treatment area increased this parameter, especially in 2017, which might also be related to the water stress caused by the purslane competition. However, some studies also reported that under-vine covering of crops had little impact on the harvest parameters of Cabernet Franc grapes, including soluble solids, titratable acidity, pH, and even the yeast assimilable nitrogen [31]. However, in other regions, where under-trellis covering crops led to moderate water stress, it increased the concentration of TSSs, which is in agreement with our present results [32].

The yield factors (fructification coefficient) and yield indicators (average cluster weight and yield per vine) of the ‘Cabernet Sauvignon’ grapes under different treatments were also surveyed. As shown in the Supplementary Materials, Table S3, there was no significant difference in the fructification coefficient between the GRASS treatment and CK in the two consecutive years, while the FILM treatment decreased it, especially in 2017. In both of the two years, the average cluster weight of the GRASS treatment grapes was slightly lower than that of CK, and the cluster weight of the FILM treatment grapes was slightly higher than CK, but no significant difference was observed. Similar trends were found in the yield per vine. Therefore, these ground treatments had little impact on the yield of grapes, which is supported by previous reports [12,27,32]. However, it was also reported that in some warmer and more arid regions, covering crops sometimes resulted in reduced vine growth and yields, while in more cool and humid regions, such as the Northeastern United States, where climates promote excessive grapevine growth, replacing bare soil with under-vine covering crops was hypothesized to improve vine growth characteristics and fruit yield by reducing excessive vigor [33]. Nevertheless, in another study of soil management conducted in a warm climate region of Southern Italy, it was also found that covering crops resulted in significantly higher yield than clean tillage, which might be due to the treatment’s effect on water availability and leaf to fruit ratio, as well as the grapevine microclimate since bud break [34].

### 3.5. Effects on Grape Aroma Compounds

A total of 54 volatile compounds in free and bound form were detected in the mature grape berries after different ground treatments, and these compounds fell into the following groups: C_6_/C_9_ compounds, higher alcohols, acids, benzenes (including volatile phenols), esters, terpenes, and C_13_-norisoprenoids, as shown in the Supplementary Materials, Tables S4 and S5. The identified aroma compounds were almost consistent with those detected in Cabernet Sauvignon grapes cultivated in Northwest China that we studied previously [9,11,15].

No matter what ground management was used, the concentration of free C_6_/C_9_ compounds, acids, and benzenes in mature grape berries was higher than that of their bound form, while that of higher alcohols, esters, terpenes and C_13_-noristoprenoids was the opposite. Because the concentration of C_6_/C_9_ compounds was much higher than that of all the other aroma substances, the total concentration of free aroma substances was much higher than that of bound aroma substances. For the two consecutive years, the concentration of total aroma compounds in the mature grape fruits of the GRASS and FILM treatments was higher than that of CK, and that of FILM was the highest. The concentration of bound aroma compounds in the mature grape berries of the GRASS treatment was significantly higher than that of CK in 2017, but the difference was not obvious in 2016. Moreover, the concentrations of the free, bond, and total volatiles in grapes in 2017 were all higher than those in 2016, respectively, no matter what ground treatments were used, which meant that the vintage meteorological difference was great enough to overcome the effects of different ground treatments.

**Figure 2:**
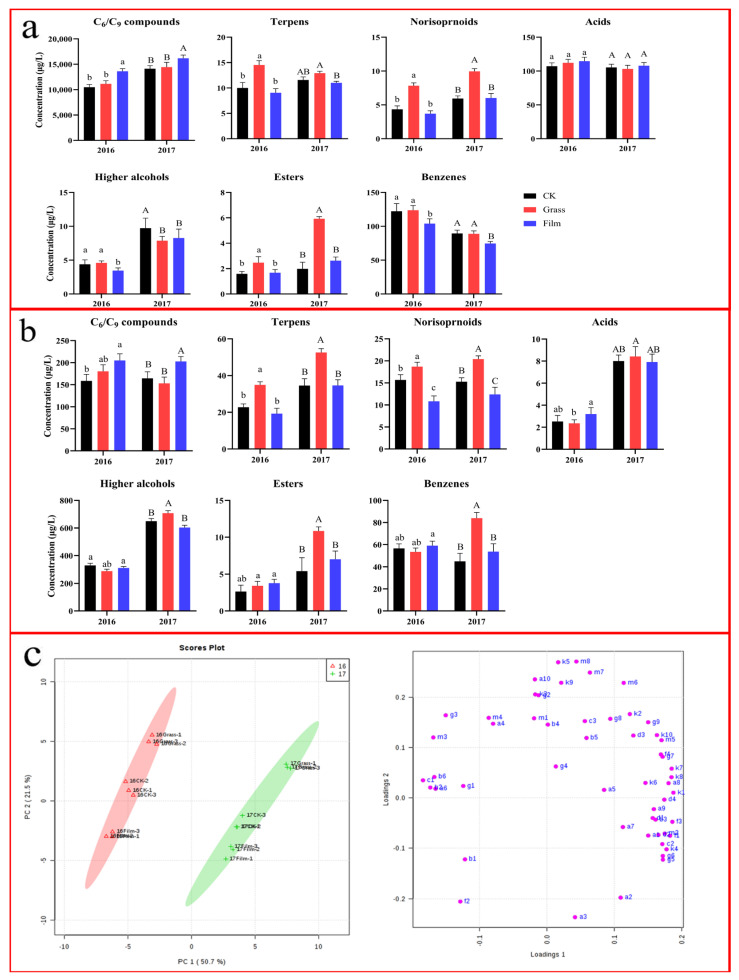
The concentrations of free (**a**) and bound (**b**) forms of aroma compounds of the mature grape berries and the PCA (**c**) under different ground management methods in the vintages of 2016 and 2017. CK: clean tillage; Grass: inner-row purslane covering; and Film: inner-row black plastic film mulching. Different letters within a vintage indicate significant differences among treatments (Duncan’s multiple range test at *p* < 0.05), the same as below. The compounds of the PCA loading plot are presented in the Supplementary Materials, Tables S4 and S5.

As shown in Figure 2a, the concentrations of various free aroma substances in the grape berries of the GRASS treatment area were generally higher than those of CK in the two consecutive years, especially terpenes, C_13_-norisoprenoids, and esters, which had significant differences, represented by geraniol, (*E*)/(*Z*)-*β*-damascenone, and geranyl acetone. This was because the purslane competed with the grapevines for water, causing slight water stress to the vines, accelerating the degradation of carotenes and the synthesis of terpenes, C_13_-norisoprenoids, and esters [35]. This was also consistent with the findings of some previous studies. For example, Kovalenko [36] found that slight water stress increased the content of terpenes in grape fruits. Feng et al. [37] found that the water deficit in the grape rhizosphere increased the accumulation of C_13_-noristoprenoids in grape fruits.

In both of the two years, the concentrations of C_6_/C_9_ compounds in the mature grape fruits of the FILM treatment area were greatly increased, while those of high alcohols and benzenes (including volatile phenols) were significantly reduced, but the impact on other classes of aroma compounds was not significant. Compared with CK, the concentrations of free terpenes and C_13_-norisoprenoids were slightly reduced and the concentrations of esters were slightly increased, but there was no significant difference. The expression of the key enzymes involved in the biosynthesis of C_6_/C_9_ compounds were often affected by the temperature, light, and water conditions [23]. The GRASS and FILM treatments changed the microenvironment of the grapevines, which led to an increase in the synthesis of C_6_/C_9_ compounds, especially in the case of the FILM treatment.

As shown in Figure 2b, GRASS had a different effect on the bound aroma compounds. GRASS significantly increased the concentrations of bound terpenes, C_13_-noriporenoids, and esters, while the concentrations of other bound aroma substances, such as C_6_/C_9_ compounds, higher alcohols, acids, and benzenes were varied between the two vintages. In 2016, the concentrations of C_6_/C_9_ compounds of GRASS were slightly higher than that of CK, but there was no significant difference, while in 2017, it was the opposite. The concentrations of higher alcohols, acids, and benzenes of GRASS were slightly lower than those of CK in 2016, but it was the opposite in 2017.

The effect of FILM on the bound aroma compounds was similar to that on the free ones. Especially, the concentrations of bound C_6_/C_9_ compounds increased significantly in both of the two vintages, thus further increasing the potential of the resultant wines’ green flavor. On the contrary, FILM reduced the concentrations of the bound terpenes, higher alcohols, and C_13_-noristoprenoids in the two years. In addition, though FILM slightly influenced the accumulation of bound esters, benzenes, and acids, no significant difference was observed. Some previous studies showed that proper light and water conditions were beneficial to the accumulation of aroma compounds in grape fruits, but too strong light, temperature, and water stress led to opposite results [29,38]. Thus, the GRASS and FILM treatments moderately adjusted the microclimate of grapevines, resulting in differences in the composition of aroma compounds.

A principal component analysis (PCA) was conducted with the total amount of all aroma compounds in the mature grapes of different ground treatments in the two consecutive years as the variables, and the results are shown in Figure 2c. In the score plot, the first two principal components explained 72.2% of the total variance, of which the principal component 1 (PC1) accounted for 50.7% of the total variance, and the principal component 2 (PC2) accounted for 21.5% of the total variance. PC1 completely discriminated between the berries of 2016 and those of 2017; the berries of 2016 were on the negative half-axis of PC1 and those of 2017 were on the positive half-axis. PC2 separated the different treatments, in which the berries of the GRASS treatment were located on the upper half axis of PC2, and those of the FILM treatment were located on the lower half axis.

According to the loading plot, most aroma compounds were close to the positive half-axis of PC1, which indicates that the concentrations of aroma compounds in grape berries of 2017 were higher than those of 2016. According to the meteorological data, the weather conditions of 2017 were more suitable for wine grape growing and winemaking than those of 2016, with less rainfall and more sunshine. Most of these substances close to the positive axis of PC1 were volatile aromatics, terpenes, and some straight-chain fatty alcohols, the concentrations of which were relatively higher in 2017 than those in 2016. The main substances on the negative half-axis of PC1 were (*E*)-2-hexen-1-al, hexanal, and other straight-chain fatty aldehydes. Most isoprenoids, i.e., (*E*)-*β*-damascenone, geranylacetone, *β*-Ionone, nerol, and cineole were located on the upper half axis of PC2. These aroma compounds with a high concentration in grape berries of the GRASS treatment area mainly contributed to the floral fragrance, which distinguished GRASS wines from CK. While 1-hexanol, (*E*)-3-hexen-1-ol, pentanol, and other substances located on the lower half axis of PC2 had higher concentrations in grape berries of the FILM treatment area, which could be used to distinguish FILM berries from other treatments.

However, in one of our previous studies of inter-row covering of crops in Northwest China, purslane growing did not only promote the accumulation of C_13_-norisoprenoids and esters in the harvest grapes but also C_6_ alcohols, which was partially different in the present study [11]. In addition, inter-row mulching also significantly increased grape C_6_/C_9_ compound concentrations, especially that of (*Z*)-3-hexen-1-ol, which had strong negative correlations with light exposure after veraison, whereas it decreased grape C_13_-noristoprenoids and terpenoids significantly, which had a strong positive correlation with low-temperature duration [15]. This finding was basically consistent with the present study.

### 3.6. Effects on Wine Physicochemical Parameters

The physicochemical parameters of the wines made from grape fruits of different ground treatments in the two consecutive years after alcoholic (AF) and malolactic fermentation (MF) were shown in Table 1. These indexes of the resultant wines showed great differences between the two vintages, since the vinification was almost the same.

After AF, the alcohol and TA concentrations of the wines obtained from each treatment of 2017 were higher than those of 2016. Correspondingly, the pH values, residual sugars, and volatile acids were lower. Although the residual sugar concentrations of the wines of 2016 were all more than 4 g/L, they were still dry red wines because the differences between the concentrations of residual sugars and total acids were all less than 2 g/L. After MF, the TA concentrations of the wines from different treatments in the two years decreased, the pH values and volatile acid concentrations increased, and the alcohol contents also slightly adjusted (increased by 0–0.3% vol).

After AF, there was no significant difference in the residual sugar concentration among different ground treatments in both of the two years. In 2016, the TA concentration of the FILM wine was the highest, which was significantly higher than that of the GRASS wine, and that of the CK wine was the lowest, which was significantly lower than that of GRASS. However, in 2017, the TA concentration of the FILM wine was still the highest, followed by that of the CK wine, and that of the GRASS wine was the lowest.

### 3.7. Effects on Wine Aroma Compounds after Alcoholic Fermentation

In the two consecutive years, the components of aroma compounds identified in the wines obtained from different ground treatments after AF are shown in the Supplementary Materials, Table S6. In total, 76 and 79 aroma compounds were identified in the wines of different ground treatments after AF in 2016 and 2017, including 5 C_6_/C_9_ compounds, 14 and 17 higher alcohols, 5 acetate esters, 15 and 16 ethyl esters, 7 other esters, 6 acids, 5 carbonyl compounds, 7 terpenes, 4 norisoprenoids, 7 benzenes (including volatile phenols), and 2 other compounds, respectively. Among them, in the wines of 2017, three alcohols and one ester were detected more than in those of 2016, namely, (*E*)-2-hexen-1-ol, 2-ethylhexanol, 3-ethyl-4-methylpentanol, and ethyl 3-hexenoate. Meanwhile, one more ester was detected in the wines of 2016 than in those of 2017, namely, isobutyl octanoate. It was observed that there was no difference in the types of aroma compounds in the same vintage, but differences in their concentrations were observed after different ground treatments.

After AF, the concentrations of the total aroma compounds (free and bound forms) in the wines had a large difference between the two vintages, which was higher in 2017 than in 2016, and significant differences were found for most aroma compounds. Exceptions occurred in the wines of CK in 2016, with terpenes concentrations higher than those in 2017. In addition, in the wines of CK and GRASS, the benzene concentrations in 2016 were higher than in 2017. It was observed that the aroma profiles of the 2017 wines were more complex and richer.

**Figure 3:**
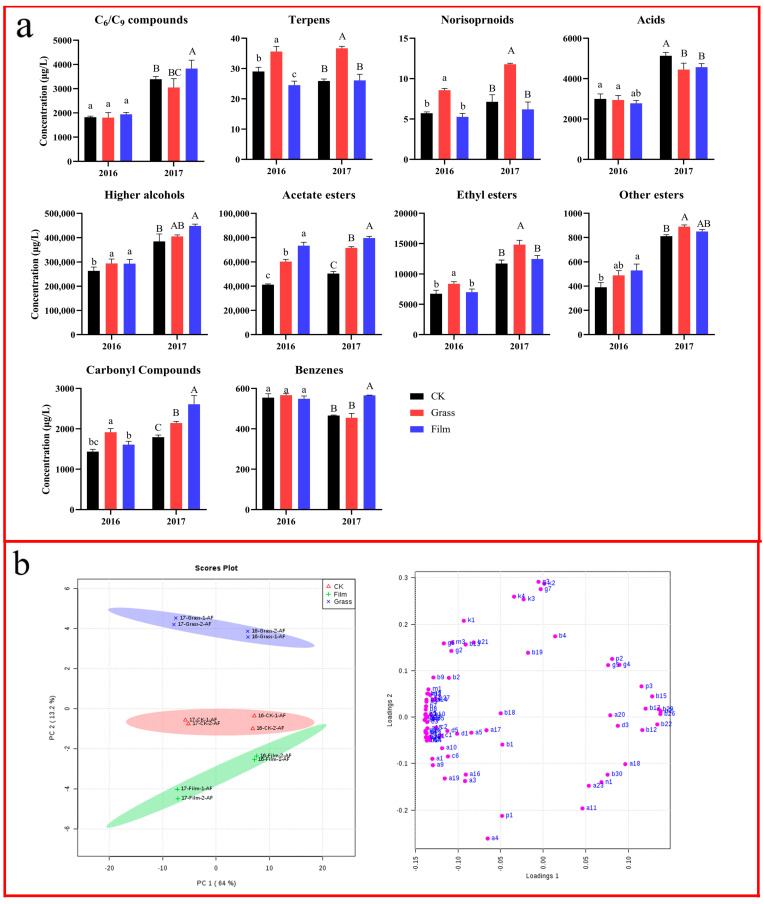
The concentrations of volatile compounds in wines after alcoholic fermentation (**a**) and the PCA (**b**) under different ground management methods in the vintages of 2016 and 2017. CK: clean tillage; Grass: inner-row purslane covering; and Film: inner-row black plastic film mulching. The compounds of the PCA loading plot are presented in the Supplementary Materials, Table S6.

As shown in Figure 3a, similar to the concentration of aroma compounds in grape fruits, those in the wines obtained from different treatments after AF in 2017 were higher than those in 2016. The concentrations of terpenes, C_13_-norisoprenoids, esters (including acetate esters, ethyl esters, and other esters), and carbonyl compounds in wines of GRASS in the two vintages were much higher than those of CK. Some previous studies also reported that crop covering promoted the production of esters in wines. For example, Xi et al. [39] found that crop covering between rows increased the concentrations of acetate esters and ethyl esters in the ‘Cabernet Sauvignon’ wines compared with clear tillage. Moreover, in the present study, the concentration of higher alcohols in the wines of GRASS was also higher than those of CK, and there was a significant difference in 2016. In addition, the GRASS treatment effectively increased the concentration of isopentanol (mellow and bitter almond flavor) and 2-phenylethanol (rose and sweet flavor), which are beneficial to the complexity and elegance of the wine aroma. Interestingly, the concentrations of C_6_/C_9_ compounds in the wines of GRASS were slightly lower than that of CK, but there was no significant difference. The concentrations of acids in the wines of GRASS were also lower than those of CK, and there was a significant difference in 2017. Because acids often produce pungent odors in wines, the moderate reduction in their concentration might be beneficial to the expression of the overall wine aroma [40]. For benzenes, the trends of the influences were different between the two vintages. Their concentration in wines from the GRASS treatment area was slightly higher than that of CK in 2016, while it was the opposite in 2017. Some reports showed that benzenes originating from the metabolism of amino acids in grape fruits, especially volatile phenols, were promoted by the water stress caused by crop covering, and their concentrations were further increased in the resultant wines [41].

Compared with CK, the GRASS treatment increased the concentrations of C_6_/C_9_ compounds in wines after AF in both of the two years, and made a significant difference in 2017, but the difference was far less than those in grape fruits, which was caused by their loss during fermentation. Similar to the impact on terpenes and C_13_-norisoprenoids in grape fruits, the FILM treatment also reduced the concentrations of these two classes of substances in the resultant wines, and caused a significant decrease in the concentration of terpenes in 2017. On the other hand, in both of the two vintages, the FILM treatment increased the concentrations of higher alcohols, acetate esters, and other esters in wines significantly, and also increased the concentration of ethyl esters but did not cause a significant difference. Furthermore, the FILM treatment also increased the concentrations of carbon compounds and benzenes in the resultant wines, especially in 2017. Compared with those of the GRASS wines, the concentrations of C_6_/C_9_ compounds, higher alcohols, acetate esters, and other esters in the wines of the FILM treatment area were higher, and the concentrations of terpenes, C_13_-norisoprenoids, and ethyl esters were lower. Moreover, the concentrations of acids, carbon compounds, and benzenes were both affected by the treatments and vintages, but the influence was inconsistent.

In the PCA of wine aroma profiles, as shown in Figure 3b, the results showed that PC1 and PC2 explained 77.2% of the total variance, of which PC1 was 64.0% and PC2 was 13.2%, respectively. In the score plot, PC1 distinguished the two vintages clearly. The wines of 2016 were located on the positive half-axis of PC1, and the wines of 2017 were located on the negative half-axis. On the other hand, PC2 separated the wines from different treatments. Among them, the wines of the GRASS treatment area were located on the upper half axis of PC2, while the wines of the FILM treatment area were located on the lower half axis. The wines of the CK area were located between those of the two treatments. This result showed that both of the vintages and the ground treatments had crucial influences on the composition of aroma substances in wines after AF. According to the loading plot, the aroma compounds located on the positive axis of PC1 were mainly other esters (isobutyl octanoate and isoamyl lactate) and several ethyl esters (ethyl 2-hexenoate, ethyl 3-hydroxybutyrate, and diethyl succinate), while the aroma substances located on the negative axis of PC1 were mainly acetate esters and other ethyl esters. This indicated that the wines of 2017 after AF had more acetate esters and ethyl esters than those of 2016.

The aroma compounds on the upper half axis of PC2 were mainly terpenes (D-limonene and linalool) and C_13_-norisoprenoids (geranylacetone, α-ionone, and β-ionone), and the aroma substances located on the lower half axis were C_6_/C_9_ compounds ((*Z*)-3-hexen-1-ol and (*E*)-2-hexen-1-ol), higher alcohols (3-methyl-1-pentanol, and (*Z*)-6-nonenol) and a sulfur compound, 3-methylthiopropanol. This suggests that the wines after AF from the GRASS treatment area contained more terpenes and C_13_-norisoprenoids, while those of the FILM treatment area contained more C_6_/C_9_ compounds and higher alcohols.

### 3.8. Effects on Wine Aroma Compounds after Malolactic Fermentation

The aroma compounds identified in wines obtained from different ground treatments after MF in the two consecutive years are shown in the Supplementary Materials, Table S7. In both of the two years, in total, 80 aroma compounds were detected, including 5 C_6_/C_9_ compounds, 17 higher alcohols, 5 acetate esters, 16 ethyl esters, 7 other esters, 6 acids, 5 carbonyl compounds, 7 terpenes, 4 C_13_-norisoprenoids, 6 benzenes, and 2 other compounds.

After MF, the concentrations of various aroma substances in the wines of 2017 were generally higher than in those of 2016, except for carbon compounds and benzenes. Compared with the wines of CK and the FILM treatment area, those of the GRASS treatment area had higher concentrations of alcohols, acetate, ethyl esters, C_13_-norisoprenoids, and volatile phenols in both of the two consecutive years. Compared with the wines of CK and the GRASS treatment area, those of the FILM treatment area had a higher concentration of C_6_/C_9_ compounds. Furthermore, the concentration of acids in the wines of CK was higher than that of the other two treatments in both of the two years. These results indicate that although MF changed the concentration of each type of aroma compound in wines, the composition was still greatly affected by the grape materials themselves.

**Figure 4:**
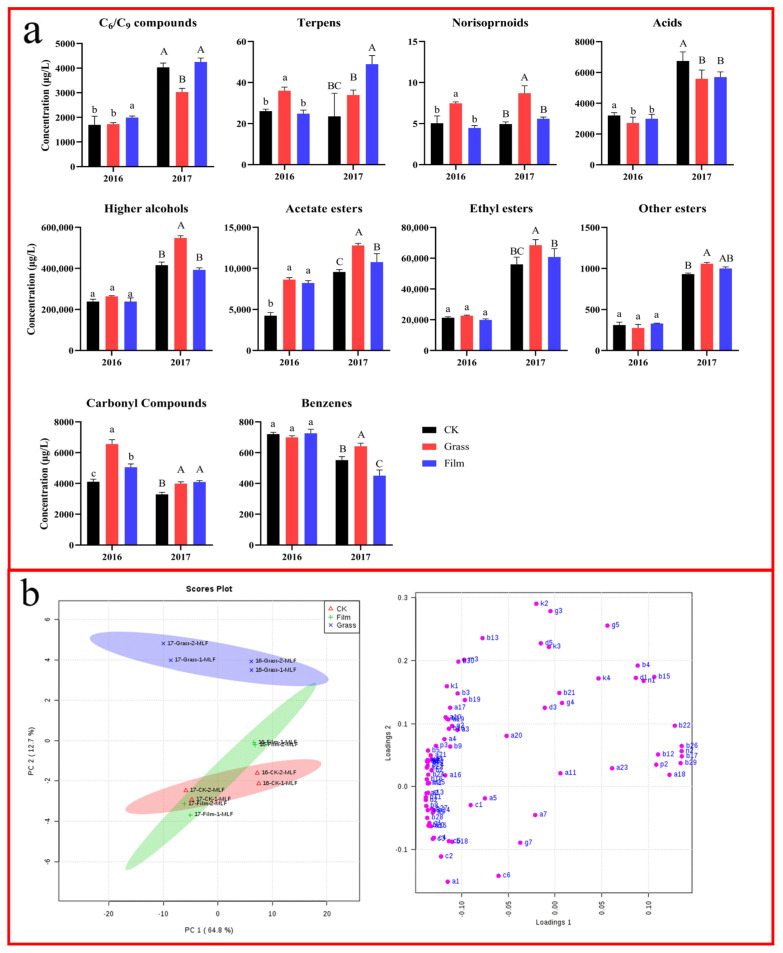
The concentrations of volatile compounds in wines after malolactic fermentation (**a**) and the PCA (**b**) under different ground management methods in the vintages of 2016 and 2017. CK: clean tillage; Grass: inner-row purslane covering; and Film: inner-row black plastic film mulching. The compounds of the PCA loading plot are presented in the Supplementary Materials, Table S7.

The PCA results of wine aroma compounds after MF of different ground treatments in the two years are shown in Figure 4b. The first two principal components cumulatively explained 77.5% of the total variance, of which PC1 explained 64.8% and PC2 explained 12.7%, respectively. It can be seen from the score plot that PC1 clearly distinguished between the two vintages. The wines of 2016 were located on the positive half-axis of PC1, and the wines of 2017 were located on the negative half-axis. However, PC2 only distinguished the wines of the GRASS treatment from those of CK and the FILM treatment, while those of the latter two groups could not be completely separated. These results show that the effects of crop covering on wine aroma compounds were still obvious after MF, but MF moderated the differences in the aroma profiles between the wines of CK and the FILM treatment area.

Though there are more and more studies conducted on soil management methods in vineyards, including covering crops or mulching, investigations about their effects on wine aromas are still quite limited, especially that of Cabernet Sauvignon [8]. In one of the previous studies on inter-row mulching, it was found that the treatment significantly decreased the concentrations of isopentanol and ethyl acetate but increased the concentrations of ethyl cinnamate, phenylacetaldehyde, phenylethyl alcohol, and 3-methylthio-1-propanol, which partially agree with the present study [15]. Besides our studies conducted in Northwest China on covering different crops or applying mulches, Xi et al. [39] studied the impact of covering crops with white clover, alfalfa, and tall fescue on the aroma profiles of Cabernet Sauvignon wines. About 57 volatile compounds were identified and quantified, and higher levels of aroma compounds were found in the wines made from the grapes with various covering crops. Through analysis, they found esters (including ethyl acetate, isoamyl acetate, ethyl octanoate, ethyl hexanoate, and phenylethyl acetate), terpenoids (including linalool, citronellol, and α-ionone) and C_13_-noristoprenoids (mainly *β*-damascenone) were the impact odorants of the wines with treatments, the contents of which were higher in the wines of covered crops than in those of clean tillage [39].

Another wine grape cultivar of interest is ‘Negroamaro’, which is an autochthonous variety of Southern Italy and is becoming more and more important to the Italian wine market. Toci et al. found that compared with permanent covering of crops, clean tillage positively affected the aroma composition of ‘Negroamaro’ wine there; indeed, higher contents of esters, carboxylic acids, alcohols, phenolics, and acetamides together with lower contents of sulfurs were detected, which were related to the higher water stress, though no substantial differences were perceived from the judges, meaning that these treatments did not have effect on sensorial profile [42,43]. A recent study conducted by the same group further validated this result [34]. However, in another study conducted in Australia in a warm climate, covering crops with Zulla in a vineyard did not produce negative effects on the volatile profiles of ‘Syrah’ must, which might be beneficial for the resultant wine aroma profiles [44]. Therefore, it was suggested that more research was needed to understand the interaction of the microclimate, the site characteristics (e.g., sloping, soil type, fertility, and water content, and depth to groundwater), and grapevine conditions (e.g., vineyard age, wine grape variety, and rootstocks) with cover-crop selection and management, which might lead to totally different results, even in wine sensory quality [8,25].

According to the loading plot, the aroma compounds located on the positive half-axis of PC1 were mainly other esters (isobutyl octanoate and isoamyl lactate) and ethyl esters (diethyl succinate and ethyl palmitate), and the aroma substances located on the negative half axis were acetate esters and most ethyl esters. These results indicate that after MF, the wines of 2017 had higher concentrations of acetate and ethyl esters than those of 2016. The main aroma substances on the upper half axis of PC2 were geranyl acetone, linalool, citronellol, and other terpenes, indicating that the wines of the GRASS treatment area still contained high concentrations of terpenes (linalool and citronellol) and C_13_-norisoprenoids (geranyl acetone) after MF, which is a major characteristic of the wines from ground treatments.

### 3.9. Odor Active Values and Aroma Profiles of Wines

To better understand the impact of different ground management methods on wine aroma performance, the active aroma values (OAVs) of each aroma compound in all the wines of the two vintages after AF and MF were calculated, as shown in the Supplementary Materials, Tables S8 and S9.

The results showed that in 2016 and 2017 there were 15 and 18 kinds of aroma substances with OAV > 1 in the wines after AF, respectively. Except for isoamyl lactate and decanal, the other aroma substances with OAV > 1 in 2017 were higher than those in 2016. In the two consecutive years, the aroma compounds with higher OAVs in the wines after AF were isoamyl acetate, ethyl caproate, ethyl decanoate, *β*-damascenone, and *β*-ionone. In addition, the OAVs of most aroma compounds in the wines of the GRASS and FILM treatment areas after AF were higher than those of CK, and that of ethyl acetate was significantly higher. Furthermore, in the wines of the GRASS treatment area, the OAVs of *β*-damascenone and *β*-ionone were significantly higher than those of the other two ground treatments.

After MF, there were 15 and 20 aroma compounds with OAV > 1 in 2016 and 2017, respectively. The aroma substances with higher OAVs in wines after MF mainly included ethyl heptanoate, phenylacetaldehyde, *β*-damascenone, and *β*-ionone. In addition, the OAVs of most aroma compounds in the wines of the GRASS and FILM treatment areas were higher than those of CK in both of the two years, especially some esters, aldehydes, and ketones, such as ethyl acetate, ethyl caproate, ethyl decanoate, nonanal, decanal, and phenylacetaldehyde. Furthermore, the wines of the GRASS treatment area had significantly higher OAVs of linalool and *β*-damascenone than wines of the FILM treatment area and CK for the two years.

**Figure 5:**
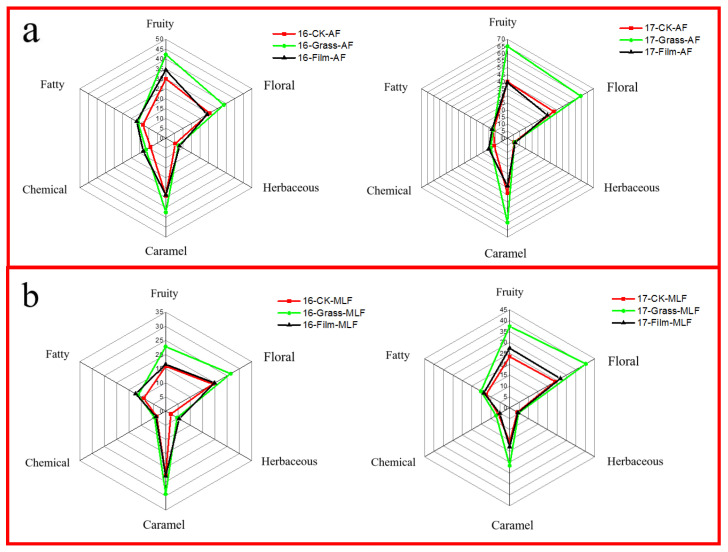
Aroma profiles of wines under different ground management methods after alcoholic fermentation (**a**) and malolactic fermentation (**b**) in the vintages of 2016 and 2017. 16: the vintage of 2016; 17: the vintage of 2017; CK: clean tillage; Grass: inner-row purslane covering; Film: inner-row black plastic film mulching; AF: after alcoholic fermentation; and MLF: after malolactic fermentation.

To more intuitively reflect the impacts of different ground management methods on the overall aroma profiles of the wines after AF and MF, aroma profile radar maps were made according to the aroma characterization of each type of aroma compound and the sum of their OAVs (Σ OAV < 1 was not listed), as shown in Figure 5. It was found that between the two vintages, the aroma contour shapes of the wines with different treatments after AF and MF were similar, but the total amount of the aroma expression had large differences. On the whole, the wine aromas of 2017 were richer and stronger. In addition, they showed more floral, fruity, and caramel aromas than those of 2016, and the wines of 2016 showed more fatty and herbaceous aromas than those of 2017.

After AF, there were significant differences in the aroma profiles of the wines of different ground management methods. Among them, the wines of the GRASS treatment area had stronger floral, fruity, and caramel flavors than those of the FILM treatment area and CK in both of the two years, while those of the FILM treatment area had more fatty, chemical, and herbaceous flavors than those of the other two ground treatments. After MF, the performance of all kinds of aromas in the wines with different treatments all decreased, among which the fruity aroma decreased the most, especially in 2016.

In the comparison of the wines of the two vintages, the aroma performance of the wines of 2017 was essentially higher than that of 2016, no matter what ground treatments were used. In 2016, the wines of the GRASS treatment area had stronger aroma performance than CK, and the performances of floral, fruity, caramel, and chemical flavors of the wines of the GRASS treatment area were also stronger than those of the FILM treatment area. In 2017, the trend was similar. Compared with the wines of CK, the wines of the FILM treatment area had more herbaceous and fatty aromas in 2016, while they had more floral, fruity, and fatty flavors in 2017.

These results are in agreement with some of our previous related studies. For example, purslane covering significantly improved the sensory value of the wines produced in Northwest China, especially the floral aroma and the complexity of the wines, and peanut growing significantly improved the sensory value of the wines, especially the aroma complexity of the wines [9,11]. On the other hand, mulching there usually led to an increase in the green aroma of the resultant wines [15].

In addition, reports from other researchers showed that covering crops or mulching had important effects on the sensory characteristics of the resultant wines. In the Northeast United States, three annual under-vine covering crops were found to significantly impact the perceived aromatic properties of ’Riesling’ wines [33]. Under-trellis covering of crops in the humid climate of Uruguay also increased the fruity aroma and the overall aroma intensity levels of the resultant ‘Tannat’ wines, which had distinctive sensory characteristics during overall palatability tests in terms of fruity aroma abundance rather than green aroma absence, which was quite similar to our results, though the climate and varieties were totally different [31]. Another study conducted in New Zealand also showed that the ‘Cabernet Sauvignon’ wines from grapes with permanent chicory covering had riper fruit aromas and flavors, as well as higher overall quality scores even after 4 years of bottle aging [45].

However, as we mentioned above, not all studies found that ground management methods (covering crops or mulching) affected wine sensory characteristics. Coletta et al. found substantial differences were perceived by judges, which meant that their clean tillage or crop covering treatment methods did not affect the wine sensory characteristics [43]. This once again showed us that the differences in terroir in various regions have a huge impact on the results of the viticultural practices of vineyard ground management methods. Without specialized local testing, growers cannot replicate the management practices and experiences of others and implement them directly, as it could lead to totally unexpected results [8,25]. Furthermore, before attempting to apply management methods, viticulturists or wine growers should consider the conditions of their grapevines, especially their variety, age, root system, etc.

## 4. Conclusions

In the region of the Eastern Foothills of the Ningxia Helan Mountains, inner-row crop covering with purslane and black plastic film mulching modifies the microclimates of the grapevines, and plays effective roles in promoting the accumulation of aroma substances in grape berries and improving the aroma performance of their wines, the effect of which was relatively stable between vintages. In comparison, inner-row crop covering especially promotes the accumulation of terpenes and C_13_-norisoprenoids in grape berries and enhances the pleasant floral and fruity fragrance in the resultant wines, which makes the aroma performance more complex and richer, while mulching mainly increases the accumulation of C_6_/C_9_ compounds that contribute to the green flavor of wines. Therefore, inner-row purslane covering in this region could certainly provide some support for the production of premium wines with better flavor and quality, and it is a ground management method for the vineyards that could be further tested and applied there. Both of the two ground management methods enhance the complexity and intensity of the wine aroma performance, and their effects were particularly notable in the vintage wines with ideal meteorological conditions; however, excessive differences in the meteorological factors between the vintages might weaken, or even mask, the impact of these two ground treatments.

## Figures and Tables

**Table 1:** Physicochemical parameters of the wines of different treatments during two stages in the two consecutive years.

Winemaking Stages	Parameters	2016			2017		
		CK	Grass	Film	CK	Grass	Film
Post alcoholic fermentation	Residual sugar (g/L)	4.5 ± 0.13 a	4.5 ± 0.17 a	4.5 ± 0.2 a	2.2 ± 0.15 ab	2.5 ± 0.01 a	2.0 ± 0.02 b
	Total acids (g/L)	5.3 ± 0.31 c	6.1 ± 0.1 b	6.5 ± 0.12 a	7.8 ± 0.52 ab	7.2 ± 0.24 b	8.0 ± 0.09 a
	pH	3.63 ± 0.21 a	3.55 ± 0.26 a	3.51 ± 0.08 a	3.45 ± 0.18 a	3.45 ± 0 a	3.45 ± 0.16 a
	Volatile acids (g/L)	0.3 ± 0 a	0.34 ± 0.01 a	0.39 ± 0.02 a	0.26 ± 0.01 a	0.23 ± 0.01 ab	0.24 ± 0.01 ab
	Alcohol (%vol)	13.8 ± 0.57 ab	14 ± 0.44 a	13.8 ± 0.79 ab	14.2 ± 0.21 a	14.3 ± 0.13 a	13.4 ± 0.12 b
Post malolactic fermentation	Total acids (g/L)	5.4 ± 0.41 a	5.8 ± 0.21 ab	5.6 ± 0.43 a	6.6 ± 0.03 ab	6.9 ± 0.16 a	6.4 ± 0.2 ab
	pH	3.86 ± 0.15 a	3.86 ± 0.23 a	3.98 ± 0.18 a	3.6 ± 0.06 a	3.59 ± 0.08 a	3.64 ± 0.24 a
	Volatile acids (g/L)	0.45 ± 0.02 a	0.39 ± 0.01 b	0.46 ± 0.01 a	0.49 ± 0 a	0.48 ± 0.03 a	0.51 ± 0.03 a
	Alcohol (%vol)	14 ± 0.56 a	14 ± 0.87 a	14.1 ± 0.13 a	14.4 ± 0.44 a	14.4 ± 1.05 a	13.6 ± 0.62 ab

All values are reported as means ± SD of three biological replicates. Different letters within a vintage indicate significant differences among berries (Duncan’s multiple range test at *p* < 0.05).

## Data Availability

The data used to support the findings of this study can be made available by the corresponding author upon request.

## Supplementary Materials

The following supporting information can be downloaded at: https://www.mdpi.com/article/10.3390/foods12132472/s1, Figure S1: Schematic diagram of different treatment cultivation of vineyard; Figure S2: Climatic parameters of the Eastern foot of Helan Mountain in 2016 and 2017 growing season; Figure S3: The influence of covering grass and film cultivation on daily average temperature, relative humidity, reflected light intensity, and water content under 40 cm of soil. Table S1: Summary of meteorological data of Helan mountain during 2016 and 2017; Table S2: The influence of covering grass and film cultivation on phenological stage; Table S3: Composing factors of productivity and yield of Cabernet Sauvignon by different cover; Table S4: Physicochemical parameters of wine in different treatments during two stages; Table S5: Concentration of free volatile compounds in the commercially harvested grape berries under cover grass and film cultivation (μg/L); Table S6: Concentration of bound volatile compounds in the commercially harvested grape berries under cover grass and film cultivation (μg/L); Table S7: Concentration of volatile compounds in different treatment wine at the end of the alcoholic fermentation and malolactic fermentation in 2016 (μg/L); Table S8: Concentration of volatile compounds in different treatment wine at the end of the alcoholic fermentation and malolactic fermentation in 2017 (μg/L); Table S9: OAVs of the aroma compounds in different treatment wine at the end of the alcoholic fermentation in 2016 and 2017; Table S10: OAVs of the aroma compounds in different treatment wine at the end of the malolactic fermentation in 2016 and 2017.
