# Supplementary material for: Effects of Inner-Row Ground Management on the Volatomics of ‘Cabernet Sauvignon’ Grapes and Wines in the Region of the Eastern Foothills of the Ningxia Helan Mountains in Northwest China
Source: Foods. 2023 Jun 23;12(13):2472. doi: 10.3390/foods12132472 (PMC10340216; doi:10.3390/foods12132472)
Supplement: Supplementary file 1 [file foods-12-02472-s001.zip › foods-2436709-supplementary/Supplementary figures.pdf]

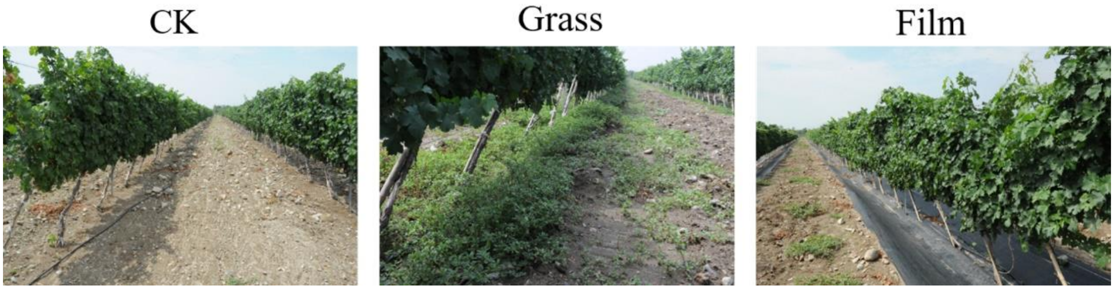

Supplementary Figure S1 Schematic diagram of different treatment cultivation of vineyard

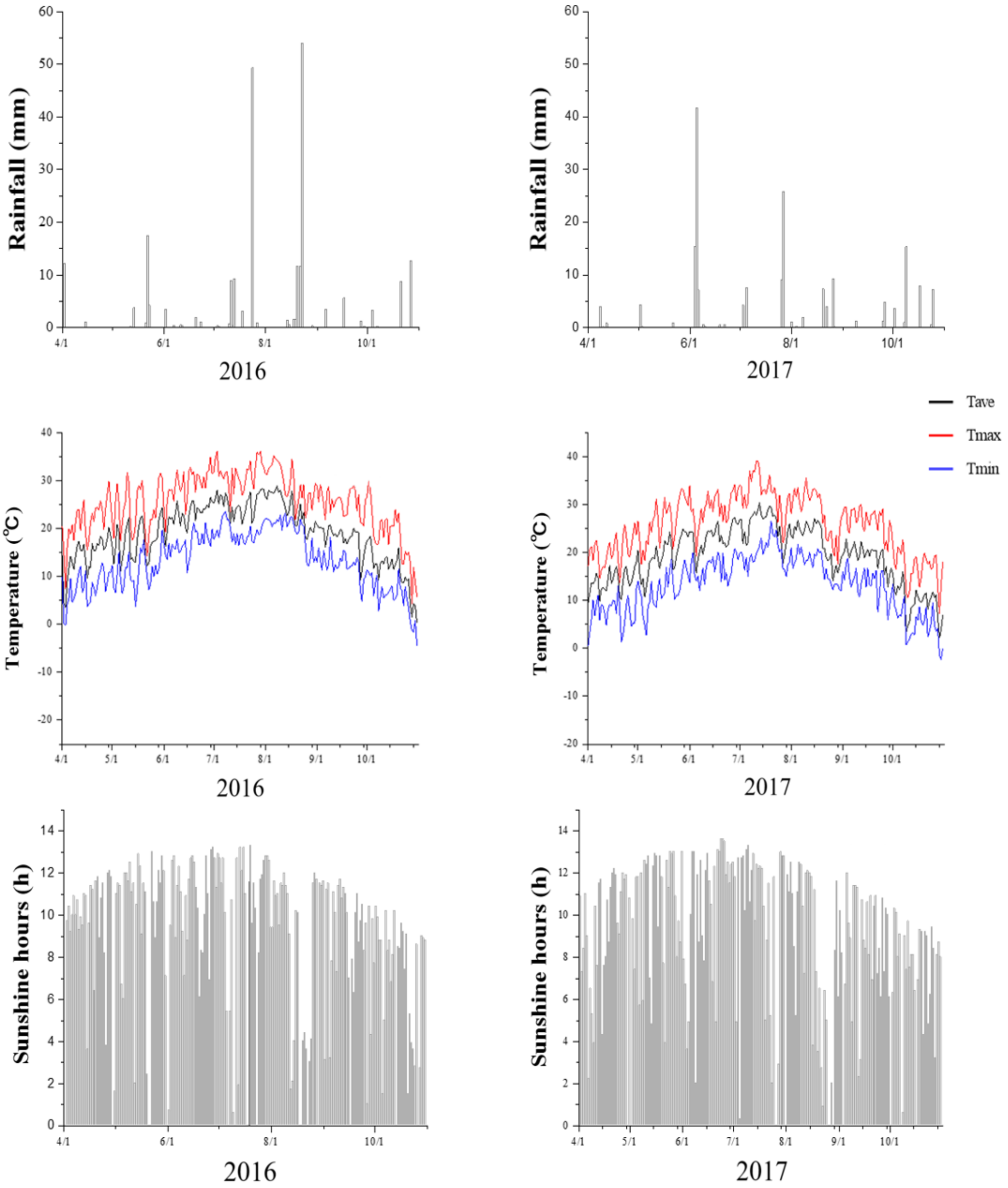

Supplementary Figure 2 Climatic parameters of the Eastern foot of Helan Mountain in 2016 and 2017 growing season

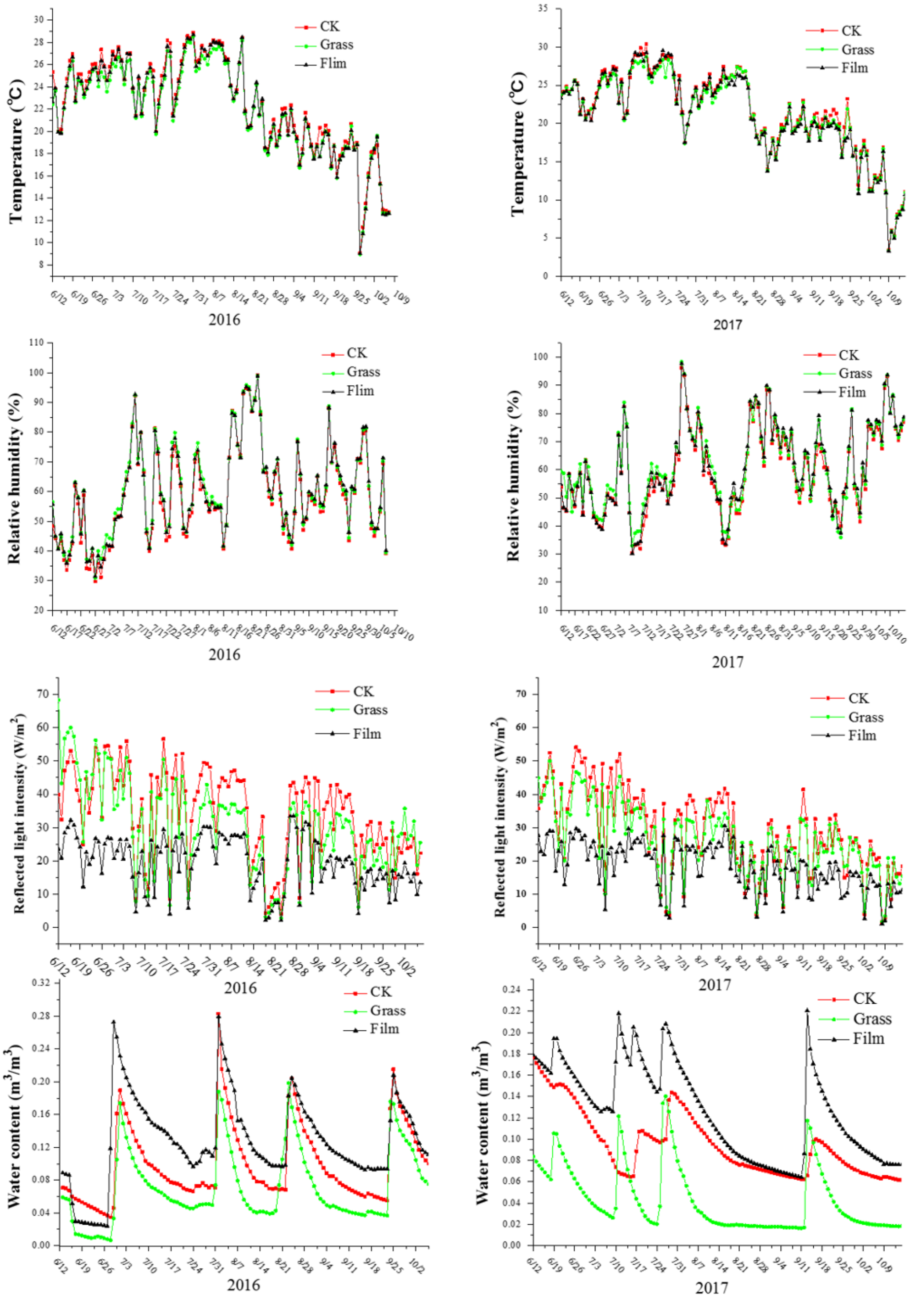

Supplementary Figure 3 The influence of covering grass and film cultivation on daily average temperature, relative humidity, reflected light intensity, and water content under 40 cm of soil
